# Monitoring Fatigue During Intermittent Exercise With Accelerometer-Derived Metrics

**DOI:** 10.3389/fphys.2019.00780

**Published:** 2019-06-26

**Authors:** Marco Beato, Kevin L. De Keijzer, Benjamin Carty, Mark Connor

**Affiliations:** ^1^ School of Science, Technology and Engineering, University of Suffolk, Ipswich, United Kingdom; ^2^ Smurfit Graduate Business School, University College Dublin, Blackrock, Ireland; ^3^ STATSports Group Limited, Newry, Ireland

**Keywords:** training, soccer, team sports, GPS, football

## Abstract

The aim of this study was to assess the sensitivity of accelerometer-derived metrics for monitoring fatigue during an intermittent exercise protocol. Fifteen university students were enrolled in the study (age 20 ± 1 years). A submaximal intermitted recovery test (Sub-IRT) with a duration of 6 min and 30 s (drill 1) was performed. In order to increase the participants’ fatigue, after that, a repeated sprint protocol (1×6 maximal 20 m sprints) was performed. Following that, participants repeated the Sub-IRT (drill 2) to evaluate the external and internal training load (TL) variations related to fatigue. Apex 10 Hz global navigation satellite system (GNSS) units were used to collect the variables total distance (TD), high metabolic distance (HMD), relative velocity (RV), average metabolic power (MP), heart rate maximal (HRmax) and mean (HRmean), muscular (RPEmus) and respiratory rating of perceived exertion (RPEres), dynamic stress load (DSL), and fatigue index (FI). A Bayesian statistical approach was used. A likelihood difference (between drill 1 and drill 2) was found for the following parameters: TD (BF_10_ = 0.33, *moderate per H_0_*), HMD (BF_10_ = 1.3, *anecdotal*), RV (BF_10_ = 0.29, *moderate per H_0_*), MP (BF_10_ = 1.3, *anecdotal*), accelerations (BF_10_ = 1.6, *anecdotal *), FI (BF_10_ = 4.7, *moderate*), HRmax (BF_10_ = 2.2, *anecdotal*), HRmean (BF_10_ = 4.3, *moderate*), RPEmus (BF_10_ = 11.6, *strong*), RPEres (BF_10_ = 3.1, *moderate*), DSL (BF_10_ = 5.7, *moderate*), and DSL•m^−1^ (BF_10_ = 4.3, *moderate*). In conclusion, this study reports that DSL, DSL•m^−1^, and FI can be valid metrics to monitor fatigue related to movement strategy during a standardized submaximal intermittent exercise protocol.

## Introduction

The quantification of training load (TL) is important for improving fitness adaptations and reducing the risk of injury in team sports (Rowell et al., 2018). A common technology utilized to quantify external TL parameters is global navigation satellite system (GNSS) (Beato et al., 2018a). A GNSS-embedded device can be used to monitor several sport-specific metrics during training sessions and matches, such as total distance covered (TD), high-speed running (HSR), and sprint running (Beato et al., 2018b). This approach consists of evaluating the TL demands by segmenting locomotion into bands based on speed thresholds. Other external TL parameters generally recorded are power-related actions (e.g., accelerations, decelerations), which may offer indications related to the neuromuscular requirements of the sport (Vanrenterghem et al., 2017). Moreover, considering the intermittent-activity profile of many team sports (e.g., soccer) (Coratella et al., 2016), heart rate (HR) should be taken into consideration, and where possible integrated with external TL variables, to exhaustively describe a players’ TL demands (Akubat et al., 2014). However, considering the nature of team sports, were high-intensity mechanical actions are generally required (Zamparo et al., 2015), recent research suggests that accelerometer-based TL variables should also be integrated with other established internal and external TL variables (Akubat et al., 2014).

Typically, GNSS-based athlete monitoring devices are also equipped with accelerometers capable of measuring linear accelerations in three axes (Barrett et al., 2016; Beato et al., 2018a). The combination of linear accelerations, known as composite acceleration, has traditionally been utilized as an external TL variable to infer the mechanical stress accumulated by an athlete (Vanrenterghem et al., 2017). The reliability of triaxial accelerometers has shown acceptable levels during running activities in team sports (Boyd et al., 2011). Moreover, the rationale related to the quantification and utilization of linear accelerations as an indicator of a player’s TL during intermittent activities has previously been reported (Barrett, 2017).

The composite derivatives of acceleration have been previously utilized to assess a player’s fatigue in team sports (Barrett et al., 2016). Fatigue has been assessed in several ways that require specific technologies (e.g., biodex) and individualized testing (e.g., isometric muscle contractions) but many team sports clubs have a limited amount of time for such assessments (Rowell et al., 2018). Metrics derived from accelerometer sensor technology during a standardized submaximal sport-specific intermittent test may detect the presence of fatigue offering the opportunity to test multiple players in an applied environment. This approach may offer further indications that could help sport scientists to understand the players’ performances over a given training period (Beato et al., 2018a). The rationale for this approach is supported by the reliability of the internal and external TL demands of a standardized test previously reported (Barrett et al., 2016). Physical variations related to transient and residual fatigue may be of interest in professional and elite levels of sport, where small differences can have a meaningful impact on performance outcomes and managerial strategies (Barrett et al., 2016; Rowell et al., 2018).

To date, information related to other accelerometer-based training load metrics used to monitor fatigue during intermittent exercises is limited; therefore, new evidence related to transient/residual fatigue monitoring using alternative algorithms may be very important for the management of players’ training loads, recovery strategies, workload implementations, and subsequent training periodization (Thorpe et al., 2015; Beato et al., 2017a). Therefore, the aim of this study was to assess several novel accelerometer-based metrics’ sensitivity to detect player’s fatigue recorded using Apex 10 Hz GNSS (STATSports, Northern Ireland) units during an intermittent standardized exercise protocol. The authors’ hypothesis is that the accelerometer-derived metrics will provide a sensitive method to assess player’s fatigue.

## Materials and Methods

### Participants and Research Design

Fifteen physically active university male students were enrolled (age 20 ± 1 years, weight 73.2 ± 6.3 kg, height 1.77 ± 0.07 m) in this study. Inclusive criteria for participation were the absence of any injury or illness by completing the Physical activity readiness questionnaire (PARQ), and regular participation in physical activity (minimum two sessions per week). Participants were asked to maintain their habitual consumption and avoid alcohol 24 h prior to exercise. Consumption of ergogenic aids was not allowed during the protocol. The experimental protocol was conducted in accordance with the Declaration of Helsinki. The Institutional Ethics Board of the University of Suffolk (Ipswich, UK) approved the experimental protocol. Written informed consent was obtained from all participants prior to participation in this study.

### Experimental Protocol and Data Analysis

Apex 10 Hz GNSS (STATSports, Northern Ireland) units were used to collect data on a synthetic outdoor pitch, in the absence of high and large buildings in the surrounding area to enhance satellite reception (Williams and Morgan, 2009). Apex units connected to a number of satellites ranging from 18 to 21 throughout the duration of the experimental protocol; the horizontal dilution of precision was 0.4 ± 0. The participants were instructed to remain in a standing position before the beginning of the experimental trials. The start time for each trial was determined by the increase above zero on the velocity trace. Participants did a familiarization trial before the beginning of the experimental procedure. Participants completed a standardized warm-up of 5 min; following that, a submaximal intermitted recovery test (Sub-IRT) with a duration of 6 min and 30 s (drill 1) was performed (Bangsbo et al., 2008). In order to increase the participants’ fatigue, after drill 1, a repeated sprint protocol (one set of six maximal 20-m sprints with 20 s of rest between repetitions) was required (Collins et al., 2018). Following the end of this protocol, participants repeated the Sub-IRT (drill 2) to evaluate the external and internal TL variations related to fatigue. For such purposes, internal TL variables should be different between drill 1 vs. drill 2 (confirming an accumulation in fatigue), while external TL variables should not be different (confirming the validity of the protocol). The rationale to use a Sub-IRT to monitoring post-match fatigue in team sports (in an ecological context) has been recently reported (Carling et al., 2018).

The Apex units were turned on 15 min before the beginning of the test, while the subjects were familiarized with the equipment as well as the protocol procedures. Apex units present the following characteristics: dimension 30 mm (wide) x 80 mm (high), weight 48 g, and 100 Hz triaxial accelerometer. Prior to the experiments, Apex unit models were placed on the back of the participant, midway between the scapula. The Apex 10 Hz is a multi-GNSS augmented unit; the validity and reliability of this unit has previously been reported (Beato et al., 2018a). GNSS data recorded by the units were downloaded and further analyzed by the STATSports Software (Apex version 2.0.2.4).

The external load variables considered in this study were: TD measured in meters, HSR that is the distance covered over 14.4 km h^−1^ (Gaudino et al., 2013), number of accelerations performed (>2 m s^−2^) (Beato et al., 2017b), and relative velocity (RV) calculated as the ratio between TD and the total time of the drill. Average metabolic power (MP) and high-intensity metabolic distance over 20 W^.^kg^−1^ (HMD) were estimated using the rationale reported in the following paper (Osgnach et al., 2010).

DSL is captured by a 100-Hz triaxial accelerometer measuring linear accelerations in the three movement axes (XYZ). Acceleration values were then aggregated over the defined period to obtain a measure of dynamic stress load using the following equation:

$$DSL\;=\;\sum_{i=1}^{n}\mathrm{IMPAC}T_{i}^{k}\;\cdot \;SF$$

where *k* is a weighting factor and *SF* a scaling factor. An *IMPACT* is defined as the maximum composite acceleration value of every 10 accelerometer points measured, if the maximum value is greater than 2G. Fatigue index (FI) is the ratio between a player’s instantaneous speed, measured by the GNSS sensor, and the linear acceleration values. Raw sensors’ readings are converted to a common scale by using a convex weighting function, similar to DSL. Therefore, FI is as follows:

$$FI_{i}=DSL_{i}/\mathrm{Spee}d_{i}^{K}$$

where *K* is a weighting factor. Reliability of accelerometer data has previously been reported (Barrett, 2017).

Participants’ internal TL was quantified using maximum HR (HRmax), which was recorded during each drill, while mean HR (HRmean) was the average HR recorded during each drill. HR was recorded during drills using Polar T31 belts (Polar, Oulu, Finland) (Beato et al., 2017b). At the end of each drill, the rating of perceived exertion (RPE) was recorded (RPE, Borg’s CR100-scale) (Impellizzeri et al., 2004). Players were asked individually to provide an RPE score in an attempt to prevent the influence of interaction with other participants’ scores. This occurred immediately after the end of each drill. RPE was quantified for both respiratory (RPEres) and muscular (RPEmus) effort (Jaspers et al., 2017). RPE construct validity in team sport was previously reported (Impellizzeri et al., 2004).

### Statistical Analyses

Statistical analyses were performed using JASP (Amsterdam, Netherland) software version 0.9.1. Data are presented as mean ± SD. The reliability between drill 1 and drill 2 was assessed using the typical error of measurement and expressed as percentage of the coefficient of variation (CV) (Atkinson and Nevill, 1998; Beato et al., 2017b). A CV(%) of less than 10% was used as a cut-off value for test–retest reliability for the following variables: TD, HMD, MP, RV, and accelerations (Atkinson and Nevill, 1998). The sample size power was calculated by G-power (tails = 2, *a* err prob. = 0.05, power = 0.8), participants = 15, estimated power = 0.82 (Wang et al., 2005). External TL variables were analyzed with a “noninformative” prior hypothesis (Cauchy, 0.707). A Bayesian statistical approach to provide probabilistic statements was used in this study; therefore, traditional inferential statistics were not reported (Sainani, 2018). A paired *t*-test was used to evaluate the difference between conditions (drill 1 vs. drill 2) on TL variables. Estimates of median standardized effect size (ES) and 95% credible interval (CI) were calculated (Ly et al., 2016). ES was interpreted by Cohen as *trivial* < 0.2, *small* 0.2–0.6, *moderate* 0.6–1.2, *large* 1.2–2.0, and *very large* > 2.0 (Cohen et al., 1990). Evidence for the alternative hypothesis (H_1_) was set as BF_10_ > 3 and evidence for null hypothesis (H_0_) was set as BF_10_ < 1/3. BF_10_ was reported to indicate the strength of the evidence for each analysis. The BF_10_ was interpreted using the following evidence categories: 1 < BF_10_ < 3 = *anecdotal* evidence for H_1_; BF_10_ ≥ 3 = *moderate*; BF_10_ ≥ 10 = *strong*; BF_10_ ≥ 30 = *very strong*; BF_10_ ≥ 100 = *extreme* (Lee and Wagenmakers, 2013).

## Results

Apex 10 Hz GNSS data recorded during drill 1 and drill 2 and reliability are reported in Table 1.

**Table 1:** Descriptive data and reliability between drill 1 and drill 2 following fatiguing protocol (*n* = 15).Data are presented as mean ± SD.

Variables	Drill 1 (SD)	Drill 2 (SD)	CV (CI 90%)	CV% (CI 90%)
TD (m)	917 ± 21	919 ± 22	7 (5; 11)	0.8 (0.6; 0.3)
HMD (m)	378 ± 49	357 ± 40	25 (19; 38)	7.8 (5.7; 12.8)
RV (m•min^−1^)	139.8 ± 5.1	140.0 ± 5.5	2.3 (1.7; 3.6)	1.8 (1.3; 2.9)
MP (W•kg^−1^)	15.8 ± 1.0	15.4 ± 0.9	0.5 (0.4; 0.8)	3.9 (2.8; 6.2)
Accelerations (n°)	33 ± 3	31 ± 4	2.3 (1.8; 3.5)	8.5 (6.2; 13.9)
HRmax (bpm)	194 ± 5	197 ± 5	2.8 (2.1; 4.5)	1.5 (1.1; 2.3)
HRmean (bpm)	172 ± 10	177 ± 7	4.3 (3.2; 6.8)	2.5 (1.9; 4.1)
RPEmus (AU)	26.7 ± 12.2	39.2 ± 20.3	9.1 (6.7; 13.8)	38.7 (27.4; 68.4)
RPEres (AU)	55.1 ± 18.3	61.1 ± 21.2	7.1 (5.4; 11.7)	12.6 (9.1; 20.7)
DSL (AU)	35.1 ± 12.7	38.1 ± 11.0	2.9 (2.2; 4.4)	7.3 (5.4; 12.0)
DSL•m^−1^ (AU)	0.038 ± 0.014	0.041 ± 0.012	0.003 (0.002;0.005)	7.6 (5.7; 12.4)
FI (AU)	0.71 ± 0.25	0.76 ± 0.22	0.06 (0.04; 0.09)	6.9 (5.0; 11.2)

SD, standard deviation; CI, confidence intervals; CV, coefficient of variation; TD, total distance; HMD, high metabolic distance; RV, relative velocity; MP, average metabolic power; HR, heart rate; RPEmus, muscular rating of perceived exertion; RPEres, respiratory rating of perceived exertion; DSL, dynamic stress load; FI, fatigue index; m, meters; min, minutes; AU, arbitrary units; bpm, beats per minute.

Bayesian analysis related to differences between drill 1 vs. drill 2 reported the following strength of the evidence: TD (BF_10_ = 0.33, *moderate per H_0_*), HMD (BF_10_ = 1.3, *anecdotal*), RV (BF_10_ = 0.29, *moderate per H_0_*), MP (BF_10_ = 1.3, *anecdotal*), accelerations (BF_10_ = 1.6, *anecdotal*), HRmax (BF_10_ = 2.2, *anecdotal*), HRmean (BF_10_ = 4.3, *moderate*), RPEmus (BF_10_ = 11.6, *strong*), RPEres (BF_10_ = 3.1, *moderate*), DSL (BF_10_ = 5.7, *moderate*), DSL•m^−1^ (BF_10_ = 4.3, *moderate), and* FI (BF_10_ = 4.7, *moderate*).

Estimates of median standardized ES and 95% CI between drill 1 vs. drill 2 for the parameters HRmax, HRmean, RPEmus, RPEres, DSL, DSL•m^−1^, and FI are reported in Table 2.

**Table 2:** Summary of baseline and post data following (drill 1 vs. drill 2) Bayesian *t*-test (*n* = 15). Data reported as standardized ES with 95% credible interval (*n* = 15).

Variables	Standardized ES	95% CI	BF_10_	Qualitative interpretation
HRmax (bpm)	0.78 *moderate*	−0.02; 1.71	2.19	Anecdotal
HRmean (bpm)	0.88 *moderate*	0.09; 1.88	4.26	Moderate
RPEmus (AU)	1.14 *moderate*	0.26; 2.03	11.59	Strong
RPEres (AU)	0.67 *moderate*	0.08; 1.47	3.13	Moderate
DSL (AU)	0.81 *moderate*	0.17; 1.64	5.71	Moderate
DSL•m^−1^ (AU)	0.74 *moderate*	0.10; 1.56	4.28	Moderate
FI (AU)	0.77 *moderate*	0.11; 1.56	4.75	Moderate

HR, heart rate; RPEmus, muscular rating of perceived exertion; RPEres, respiratory rating of perceived exertion; DSL, dynamic stress load; FI, fatigue index; BF_10_, Bayesian factor; ES, effect size; CI, credible intervals; AU, arbitrary units; bpm, beats per minute.

## Discussion

The aim of this study was to assess the sensitivity of novel accelerometer-based metrics (e.g., DSL) at detecting player’s fatigue recorded using 10 Hz GNSS units, with an embedded accelerometer, during an intermittent submaximal standardized exercise protocol. This study is the first to report that DSL, DSL•m^−1^, and FI can be valid metrics for monitoring fatigue-related modifications to movement strategies during a standardized submaximal intermittent exercise protocol. Therefore, DSL, DSL•m^−1^, and FI could be utilized by sport practitioners as valid metrics for the management of fatigue (post-match and post-training fatigue) and TL monitoring in sports.

The main findings of this research were that the external TL variables (e.g., TD, RV, MP) did not change between drill 1 vs. drill 2 (Table 1), while internal TL demands increased in drill 2 vs. drill 1, which support the validity of the protocol (Table 1 and Table 2). Subsequently, accelerometer-based metrics (e.g., DSL, DSL•m^−1^, and FI) increased in drill 2 vs. drill 1, demonstrating sensitivity when monitoring physical variations related to fatigue. These results may have great applicability in professional team sports, where such player’s fatigue variations may have an impact on fitness adaptations and injury risk reduction (Barrett et al., 2016; Vanrenterghem et al., 2017; Rowell et al., 2018). The validity of accelerometer measurements as a metric used in team sports to accurately and objectively quantify players’ TL has previously been reported and the results of the current study support such findings (Barrett et al., 2016; Barrett, 2017; Beato et al., 2017a; Rowell et al., 2018). Moreover, DSL showed better correlation with internal TL variables than TD, MP, and high-intensity running (Casamichana et al., 2013; Sparks et al., 2017). These results suggest that DSL may be an effective metric to take into consideration in team sports and should be integrated with more traditional internal and external TL variables. Moreover, considering that many players do not wear HR monitors during official games, DSL monitored using GNSS-embedded accelerometers may be a valid indicator of fatigue as has been recently reported (Sparks et al., 2017). DSL may be particularly important in an ecological context because these metrics are not affected by external temperature, players’ mental fatigue, and hydration status, while HR has shown to be affected by these factors (Greig et al., 2006; Thorpe et al., 2016).

A limitation related to this study is the sample utilized that is composed of physically active university male students. Therefore, the results reported in the current study should be considered carefully when elite and professional football players are monitored since fatigue may be associated with physical activity level and sport specificity. This is supported by previous evidence that showed a different energy cost between sporting populations during intermittent running exercises (Zamparo et al., 2015).

In conclusion, accelerometer-based metrics like DSL and FI are sensitive for monitoring player’s fatigue during a submaximal intermittent exercise protocol. Future studies could replicate the current study involving elite and professional players to clarify if such metrics may be valid when monitoring fatigue in a sport-specific population.

## Practical Applications

DSL can be used as an external TL metric with confidence when monitoring team sport athletes (e.g., during training sessions and matches). Practitioners may evaluate DSL, DSL^.^m^−1^, and FI by GNSS-embedded accelerometers during a Sub-IRT in order to monitor in a valid and quick way (throughout the season) athletes’ fatigue. This approach may offer further support to practitioners’ decision-making process.

## Author Contributions

MB and MC have designed the study. MB, KD, and BC have recorded the data. MB and MC have performed the data analysis. MB, KD, BC, and MC have written the paper.

### Conflict of Interest Statement

MC was employed at the time of the study by STATSports.

The remaining authors declare that the research was conducted in the absence of any commercial or financial relationships that could be construed as a potential conflict of interest.
